# Digitally-Driven Surgical Guide for Alveoloplasty Prior to Immediate Denture Placement

**DOI:** 10.3390/dj13080333

**Published:** 2025-07-22

**Authors:** Zaid Badr, Jonah Jaworski, Sofia D’Acquisto, Manal Hamdan

**Affiliations:** 1 Technological Innovation Center, Department of General Dental Sciences, School of Dentistry, Marquette University, Milwaukee, WI 53233, USA; 2 Predoctoral Program, School of Dentistry, Marquette University, Milwaukee, WI 53233, USA; 3 Department of Surgical and Diagnostic Sciences, School of Dentistry, Marquette University, Milwaukee, WI 53233, USA; manal.hamdan@marquette.edu

**Keywords:** alveoloplasty, additive manufacturing, surgical guide, immediate denture

## Abstract

**Objective:** This article presents a step-by-step digital technique for fabricating a 3D-printed surgical guide to assist in alveoloplasty for immediate denture placement. **Methods:** The workflow integrates intraoral scanning, virtual tooth extraction, digital soft tissue modeling, and additive manufacturing to produce a customized guide with an occlusal window and buccal slot, along with a verification stent. **Results:** This method ensures precise ridge recontouring and verification, enhancing surgical predictability and prosthetic fit. **Conclusions:** Unlike traditional surgical guides based on conventional casts or manual fabrication, this fully digital approach offers a practical and replicable protocol that bridges digital planning and clinical execution. By improving surgical precision, reducing operative time, and ensuring optimal denture fit, this technique represents a significant advancement in guided pre-prosthetic surgery.

## 1. Introduction

Alveoloplasty is a routine pre-prosthetic procedure aimed at creating a smooth and even alveolar ridge for optimal denture seating [1]. Traditional methods rely on intraoperative judgment, which can lead to unpredictable outcomes [2]. Recent advances in digital dentistry, including intraoral scanning, computer-aided design (CAD), and additive manufacturing, have enabled the development of surgical guides that facilitate precise bone surgery and prosthesis fabrication [3,4].

The need for alveoloplasty during immediate denture fabrication arises from the necessity to create a stable and uniform ridge that accommodates the prosthesis without causing discomfort or compromising retention [5]. Uneven ridges [6], sharp bony projections [7], or irregular tissue contours [8] can lead to pressure points, poor fit, and long-term complications for the patient [9]. By performing alveoloplasty, clinicians can ensure an optimal foundation for the immediate denture, allowing for improved function, esthetics, and patient comfort [10]. This procedure is particularly crucial in cases where multiple extractions have resulted in significant ridge irregularities.

Conventional surgical guides, when used, are often fabricated manually using physical diagnostic casts, which pose several clinical and logistical challenges [11]. These methods depend on conventional impressions that are prone to distortion, especially during transport or extended storage, thereby compromising accuracy [12,13]. In patients with severe gag reflexes or advanced periodontal disease with mobile teeth, obtaining accurate impressions can be difficult or even impossible [14,15]. Additionally, simulating surgery on physical casts is time-consuming and lacks reproducibility [16]. Moreover, traditional mock surgeries performed on stone casts are non-reversible, requiring complete repetition if errors occur or the cast breaks, leading to inefficiency and variability in outcomes. Therefore, the resulting guides often lack consistency and duplicating them for future use or documentation is not easily feasible [17].

The 3D-printed surgical guides provide an effective way to streamline procedures by combining digital planning and additive manufacturing [18]. Previous studies have highlighted the benefits of digital workflows in dental procedures, including improved accuracy [19], reduced operative time [20], and better patient outcomes [21]. This technique utilizes two CAD software programs and a 3D-pinter to design and fabricate a surgical guide for alveoloplasty prior to immediate denture placement. By replacing conventional impressions and physical mock surgeries with digital intraoral scanning and virtual design, this approach eliminates errors caused by impression distortion, cast breakage, and manual trimming [22,23]. The use of CAD software allows for precise planning and reproducible modifications, while 3D printing ensures consistent guide fabrication, reducing variability and enabling rapid turnaround. Together, these tools offer a more efficient, accurate, and scalable solution compared to traditional methods [11,16,22].

## 2. Materials and Methods

**Figure 1:**
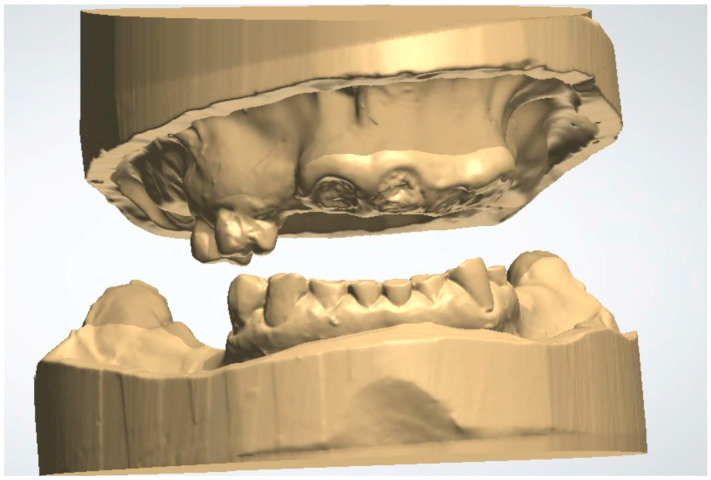
Pre-operative maxillary and mandibular digital impressions imported into 3Shaped Dental Manager.

**Figure 2:**
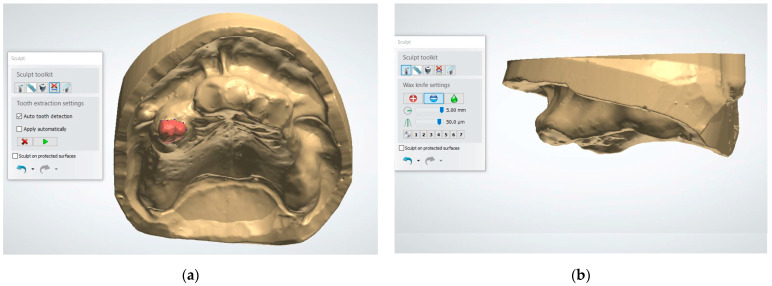
Using the Tooth Removal Tool: (**a**) the tooth to be virtually extracted is selected; (**b**) the tooth is virtually extracted after selection.

**Figure 3:**
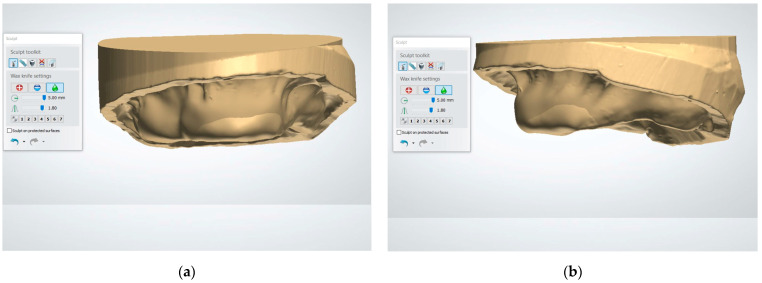
The virtual alveolopasty procedure using the Wax Knife tool to remove the undercuts and irregular ridge contours: (**a**) frontal view; (**b**) lateral view.

**Figure 4:**
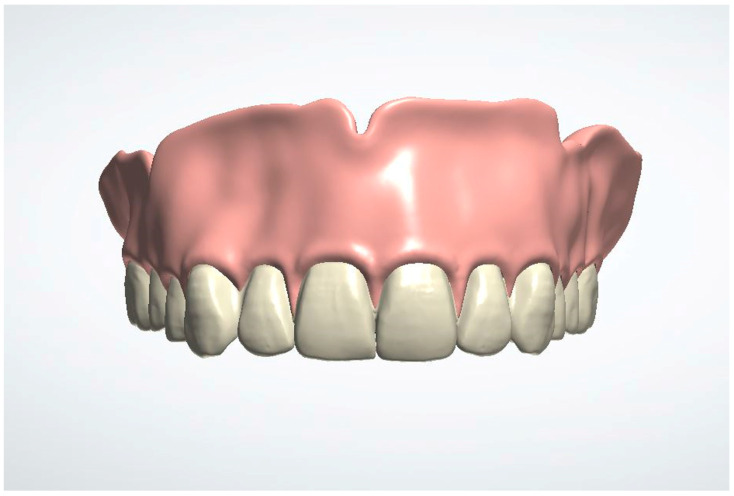
The immediate denture is designed based on the new maxillary arch contours.

**Figure 5:**
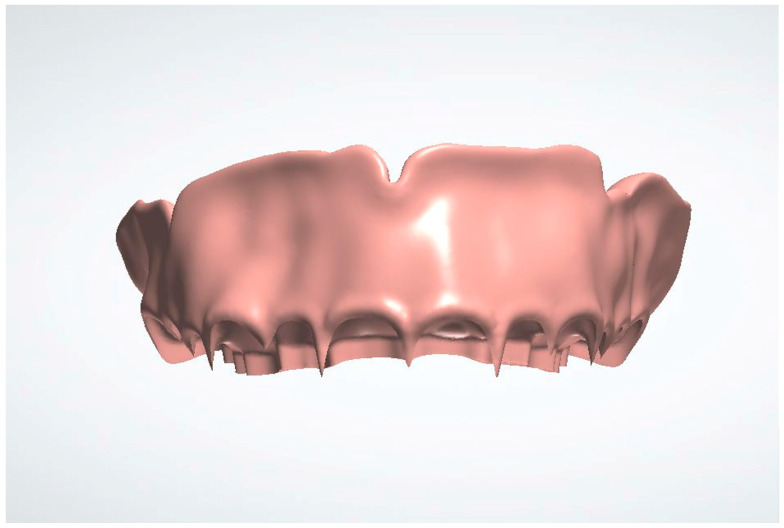
The denture base of the immediate denture design is segmented and exported in STL format.

**Figure 6:**
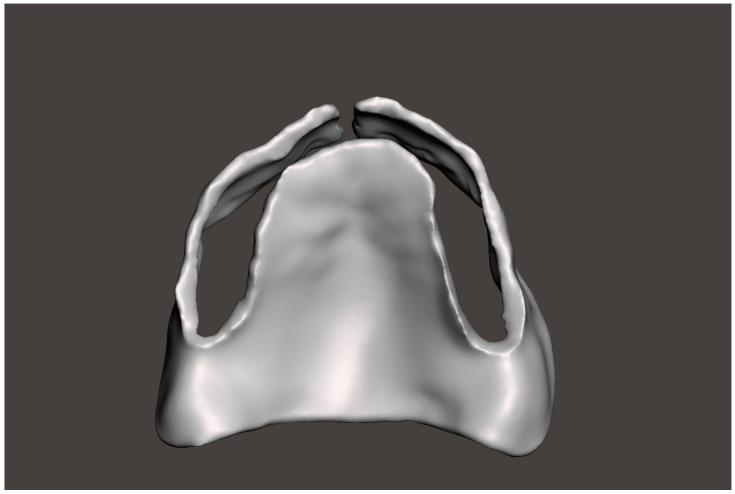
The denture base of the immediate denture design is used to design the surgical guide using Autodesk Meshmixer. The surgical guide is designed with a buccal cut and occlusal slot.

**Figure 7:**
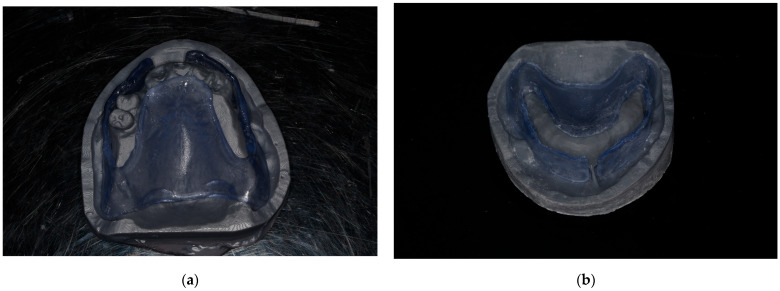
3D-printed surgical guide: (**a**) before adjustment, the flexibility of the selected material allows for the insertion of the guide underneath the undercut; (**b**) after adjustment, the width of the anterior slot is used to verify adequate buccal ridge augmentation.

**Figure 8:**
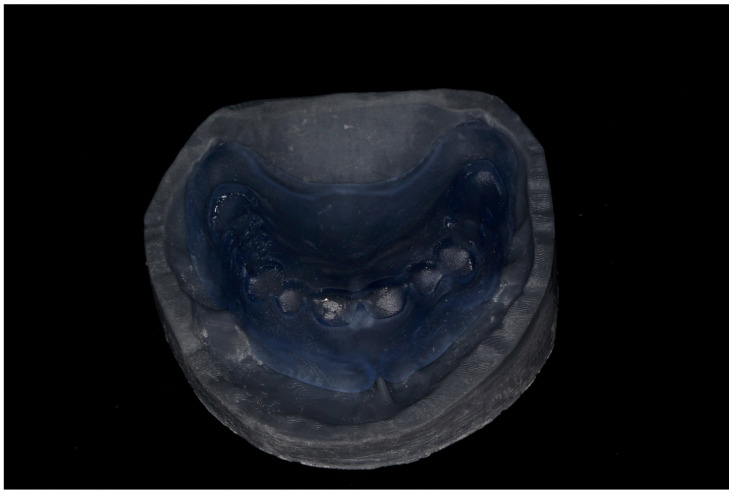
A 3D-printed verification stent is used to verify the proper ridge contours.

1. Import the digital impressions of the patient’s pre-operative maxillary and mandibular arches into a dental CAD software (3Shape Dental System version DS2022-1/2.22.1.0 or later; 3Shape A/S, Copenhagen, Denmark) for the fabrication of a complete denture (Figure 1). The digital impressions could either be direct intraoral scans, or laboratory scans of stone casts or analog impressions.

2. Use the “Tooth Removal” tool to virtually extract the teeth, simulating the post-extraction ridge anatomy (Figure 2). This tool allows the user to define the long axis of each tooth and segment it from the digital cast. Once removed, the software automatically fills the socket area by generating a smooth surface over the extraction sites based on the contours of the adjacent gingiva. This virtual soft tissue approximation simulates the anticipated post-extraction ridge morphology. However, some bony projections may still remain visible after applying this tool. To ensure a more accurate and clinically appropriate representation of the post-extraction ridge, it is essential to carefully inspect the auto-generated model and manually refine the contours when necessary.

3. Use the “Wax Knife” tool to digitally recontour the alveolar ridge. Create a smooth and contoured ridge surface, simulating the expected results of alveoloplasty (Figure 3). The Wax Knife tool offers two primary functions, remove and smooth, that allow for precise digital recontouring of the alveolar ridge. The remove function allows the user to selectively subtract excess material or virtual bony peaks that may protrude from the auto-generated surface, effectively mimicking surgical bone reduction. This is particularly helpful in areas where residual ridges appear irregular or sharp, which could interfere with prosthetic planning or guide stability. The smooth function, on the other hand, helps to blend and polish the surrounding areas by softening transitions and eliminating minor surface irregularities. This function is beneficial in creating a continuous, contoured ridge that mirrors the expected results of an alveoloplasty procedure. By alternating between the remove and smooth functions, clinicians can create a virtual ridge form that is both anatomically realistic and surgically feasible.

4. Design the immediate denture (Figure 4). The recommended software settings for denture base design are as follows: use the natural wax template; set a 0.10 mm space between the denture base and the soft tissue; ensure a minimum thickness of 0.5 mm beneath the teeth; enable the “Remove Undercuts from Teeth Pockets” feature; and disable the “Drill Compensation of Teeth Pockets” option.

5. Export the denture base in Standard triangle language (STL) format for modification (Figure 5).

6. Import the STL file of the denture base into a universal CAD software (Meshmixer version 3.5; Autodesk, San Francisco, CA, USA). Create an occlusal slot to guide the surgical reduction. Add buccal cut to allow the seating of the guide during surgery. Use the buccal flange to guide and verify the buccal surgical reduction (Figure 6). Design these features using the following steps: First, use the “Select” tool to define the areas for the occlusal window and buccal slot. For the occlusal window, select the region corresponding to the teeth sockets on the denture base. For the buccal slot, extend the selection vertically from the midline of the occlusal window to the full depth of the denture base flange. Next, use the “Edit—Erase & Fill” tools to perform the initial cuts. Finally, smooth all transitions using the “Sculpt—Robust Smooth” tool, with a strength setting of 15–20%.

7. Export the modified STL file for additive manufacturing (Pro 95s; Sprintray, Los Angeles, CA, USA). Import it into the computer-aided manufacturing (CAM) software (RayWare software; version 2.8 or later; Sprintray, Los Angeles, CA, USA). Select (NG Flex, Sprintray, Los Angeles, CA, USA) as the material. Set the printing orientation at 30–45° with supports placed on non-critical surfaces. Use the following parameters on the 3D printer (Pro 95s; Sprintray, Los Angeles, CA, USA): Layer Thickness: 100 microns, Support Density: 80%, Tip Size: 0.6 mm. Use a biocompatible flexible translucent material to fabricate the surgical guide (NG Flex, Sprintray, Los Angeles, CA, USA) to allow the insertion underneath the undercut (Figure 7). Ensure the guide is free from defects and fits accurately. Fabricate a duplicate of the unmodified denture base STL file using the same settings and material to serve as verification stent.

8. Use the verification stent to confirm proper ridge contours after alveoloplasty and before immediate denture placement (Figure 8).

**Figure 9:**
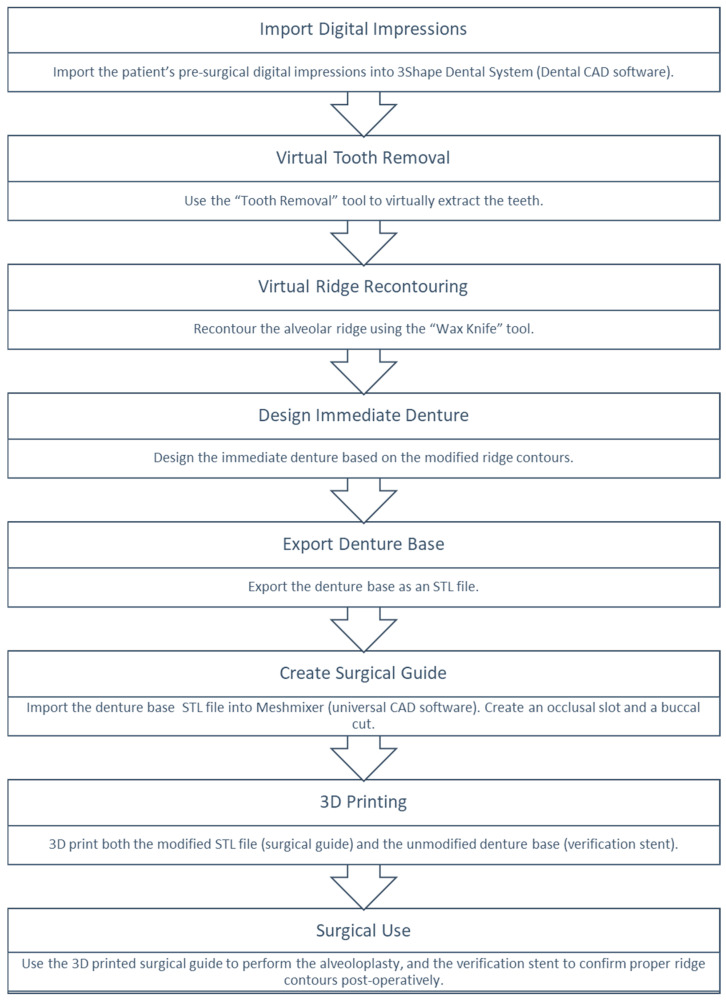
Flowchart summarizing the digital workflow for designing and fabricating the surgical guide and verification stent.

A summary of the technique is presented in (Figure 9) as a flowchart illustrating the digital workflow for the design and fabrication of the surgical guide and verification stent.

## 3. Results

The digital technique described results in well-fitting interim dentures that require minimal intraoperative adjustments. The use of the 3D-printed surgical guide allowed for precise alveoloplasty, ensuring the elimination of undercuts and sharp bony edges, which contributed to improved comfort and tissue adaptation of the immediate prosthesis. Compared to conventional freehand methods, the digital workflow offered superior surgical precision, reduced chair time, and greater predictability in prosthetic fit. Additionally, the verification stent provided an effective means to confirm ridge contours before denture delivery, further enhancing clinical efficiency and patient satisfaction.

## 4. Discussion

Digital workflows offer numerous advantages that extend beyond individual procedures, fundamentally transforming how dental care is delivered. By enabling digital data acquisition, virtual treatment planning, and CAD/CAM fabrication, digital workflows enhance the accuracy, efficiency, and reproducibility of clinical outcomes across various specialties, including prosthodontics, implantology, and oral surgery [24]. These workflows facilitate the communication between the surgical and prosthetic phases, allow for easier record keeping and retrieval, and support the creation of patient-specific solutions [25]. Furthermore, digital tools improve visualization and diagnostics, empower interdisciplinary collaboration, and enable simulations that were previously difficult with analog methods [26]. Collectively, these advantages contribute to a more predictable and patient-centered approach to care.

As these benefits become more evident, there is a growing trend among clinicians to incorporate digital workflows into routine practice. Recent surveys and market data reflect a steady increase in the use of intraoral scanners, digital treatment planning software, and chairside 3D printers in both academic and private practice settings [27,28]. Dental education programs are also adapting, integrating digital dentistry into curricula to prepare future clinicians for technology-driven practice [29,30]. This shift is not merely a response to technological innovation, but a reflection of changing patient expectations and economic pressures that demand efficiency without compromising quality. While the transition requires an investment in equipment and training, many practitioners report improved workflow integration, enhanced diagnostic capabilities, and increased patient acceptance once digital systems are fully implemented [31].

The application of this digitally guided alveoloplasty technique is particularly advantageous for patients undergoing full-mouth extractions with immediate denture placement. In such cases, achieving a smooth, evenly contoured ridge is essential for ensuring optimal prosthesis adaptation, comfort, and stability. Traditional approaches rely heavily on the clinician’s intraoperative judgment, which often leads to inconsistent outcomes and requires time-consuming adjustments or post-delivery reline [32]. The ability to digitally plan and fabricate a patient-specific surgical guide enhances precision in ridge recontouring and standardizes surgical results across operators and cases [33].

A major clinical benefit of this approach is the significant reduction in chair time for both the patient and the clinician. Digital planning allows for preoperative visualization of the post-extraction ridge morphology, guiding the surgical process to target only the necessary areas of bony reduction. As a result, intraoperative trial-and-error is minimized, and the surgeon can proceed with greater confidence and efficiency. The occlusal window and buccal slots incorporated into the guide facilitate accurate seating and visual verification during surgery, while the verification stent provides a tangible tool to confirm the adequacy of alveoloplasty before the delivery of the immediate prosthesis. This staged approach improves the predictability of prosthetic outcomes and contributes to greater patient satisfaction.

Despite its benefits, this technique has inherent limitations. One notable challenge lies in its application to the mandible. In the maxilla, the hard palate provides a stable and consistent reference surface, allowing the surgical guide to seat securely and resist displacement during bone contouring [34]. In contrast, the mandible lacks such a reference, which may compromise guide stability and accuracy during the procedure [35]. While the verification stent still serves a valuable role in confirming proper ridge contour post-operatively, the initial accuracy of bone reduction may be less predictable in mandibular applications unless additional stabilization techniques are used (e.g., fixation pins). In these cases, clinicians may also consider extending the flange design or incorporating fixation holes to improve guide retention. If guide movement occurs intraoperatively, stopping to confirm proper seating before proceeding with reduction is critical. Printing a duplicate guide as a backup may help in case of contamination obstructing visibility or fit discrepancies.

Another limitation is accessibility. The technique requires access to advanced digital tools and equipment, including intraoral scanners, CAD/CAM software, and a high-resolution 3D printer. Although the cost of digital equipment has decreased in recent years, the initial investment may still be a barrier for some private practices or institutions, particularly those in resource-limited settings [36]. Furthermore, clinicians and auxiliary staff must acquire and maintain proficiency with these digital platforms, which involves a learning curve and ongoing continuing education [37]. Successful implementation of such a technique requires cross-disciplinary collaboration between surgical, prosthodontic, and digital dental teams. Common challenges during early adoption include difficulties in mastering digital design software, such as the virtual extraction and wax knife tools in 3Shape or STL modifications in Meshmixer [38]. These steps require attention to anatomical detail and familiarity with interface functionality. Missteps in file editing can result in incomplete guide coverage, poor adaptation to the ridge, or inadequate window placement for visualization. To mitigate these risks, clinicians are encouraged to complete practice designs on non-clinical cases, attend hands-on workshops, or collaborate with experienced digital labs or clinicians [39].

Troubleshooting should also account for intraoperative issues. If the surgical guide does not fully seat, clinicians should evaluate for unrelieved undercuts, soft tissue impingement, or errors in the virtual model. Gentle intraoral adjustments or soft tissue displacement with gauze or retractors may help improve seating. If the guide appears clinically inaccurate despite proper seating, verify that the correct print file was used and that the guide was cured properly. In the event that the verification stent does not sit after alveoloplasty, it may indicate under-reduction or bony interference. In such cases, spot-reducing using a surgical handpiece while repeatedly testing the stent can help restore alignment to the planned outcome.

Clinical scenarios with extreme ridge irregularities, pronounced exostoses, or complex anatomical variations may also pose limitations to this digital workflow. In such cases, virtual simulations may not accurately predict soft and hard tissue behavior during or after alveoloplasty. This could necessitate intraoperative adjustments despite pre-planned guidance, reducing the technique’s overall efficiency. However, when used as part of a hybrid approach combining digital and conventional verification methods, the workflow remains valuable for most routine and moderately complex immediate denture cases. Ultimately, the key to successful integration lies in combining thoughtful pre-surgical planning, proper team training, and contingency protocols to address any deviations from the digital simulation during the clinical procedure [37,38,39].

In summary, the digital workflow for fabricating a 3D-printed surgical guide for alveoloplasty and immediate denture placement represents a meaningful advancement in prosthodontic surgery. It addresses key limitations of traditional freehand methods by improving precision, reducing chair time, and standardizing clinical outcomes. The ability to preoperatively simulate ridge reduction and fabricate a corresponding surgical guide improves interdisciplinary communication and case predictability. With continued advances in material science, software development, and clinician training, this approach is expected to become more widely adopted and accessible across varied clinical settings.

## 5. Conclusions

This article describes a digital workflow for fabricating a 3D-printed surgical guide for alveoloplasty prior to immediate denture placement. The technique ensures accurate ridge recontouring, reduces operative time, and improves prosthetic outcomes. By combining digital impressions, CAD tools, and additive manufacturing, this method represents a significant advancement in pre-prosthetic surgery.

## Data Availability

No new data were created.

## Abbreviations

The following abbreviations are used in this manuscript:

CAD	Computer-aided design
CAM	Computer-aided manufacturing
STL	Standard triangle language
NG	Night guard
3D	Three-dimensional

## References

[1] Dean O.T. (1936). Surgery for the denture patient. J. Am. Dent. Assoc..

[2] Hillerup S. (1994). Preprosthetic surgery in the elderly. J. Prosthet. Dent..

[3] Schweiger J., Edelhoff D., Güth J.F. (2021). 3D Printing in Digital Prosthetic Dentistry: An Overview of Recent Developments in Additive Manufacturing. J. Clin. Med..

[4] Nagata K., Fuchigami K., Hoshi N., Atsumi M., Kimoto K., Kawana H. (2021). Accuracy of guided surgery using the silicon impression and digital impression method for the mandibular free end: A comparative study. Int. J. Implant. Dent..

[5] Bourgoyne J.R. (1951). Alveoloplasty in preparation for the immediate denture insertion. J. Prosthet. Dent..

[6] Komagamine Y., Kanazawa M., Sasaki Y., Sato Y., Minakuchi S. (2017). Prognoses of new complete dentures from the patient’s denture assessment of existing dentures. Clin. Oral Investig..

[7] Hyde T.P. (2003). Case report: Differential pressure impressions for complete dentures. Eur. J. Prosthodont. Restor. Dent..

[8] Kelly E. (1972). Changes caused by a mandibular removable partial denture opposing a maxillary complete denture. J. Prosthet. Dent..

[9] Crawford R.W., Walmsley A.D. (2005). A review of prosthodontic management of fibrous ridges. Br. Dent. J..

[10] Meyer I. (1966). Alveoloplasty—The oral surgeon’s point of view. Oral. Surg. Oral. Med. Oral. Pathol..

[11] Cho S.H., Schaefer O., Thompson G.A., Guentsch A. (2015). Comparison of Accuracy and Reproducibility of Casts Made by Digital and Conventional Methods. J. Prosthet. Dent..

[12] Ahmad R., Shabestari G.O., Zeighami S., Samadi M.J., Shamshiri A.R. (2014). Effect of Storage Time of Extended-Pour and Conventional Alginate Impressions on Dimensional Accuracy of Casts. J. Dent..

[13] Punj A., Bompolaki D., Garaicoa J. (2017). Dental Impression Materials and Techniques. Dent. Clin. N. Am..

[14] Puryer J., Forbes-Haley C., Blewitt I. (2015). Dental Management of the ‘Gagging’ Patient: Challenges and Strategies. Int. J. Dent. Health Sci..

[15] Yildirim-Bicer A.Z., Akarslan Z.Z. (2014). Influence of Gag Reflex on Removable Prosthetic Restoration Tolerance According to the Patient Section of the Short Form of the Gagging Problem Assessment Questionnaire. J. Adv. Prosthodont..

[16] Stromeyer S., Wiedemeier D., Mehl A., Ender A. (2021). Time Efficiency of Digitally and Conventionally Produced Single-Unit Restorations. Dent. J..

[17] Abduo J. (2019). Accuracy of Casts Produced from Conventional and Digital Workflows: A Qualitative and Quantitative Analyses. J. Adv. Prosthodont..

[18] Wang X., Mu M., Yan J., Han B., Ye R., Guo G. (2024). 3D printing materials and 3D printed surgical devices in oral and maxillofacial surgery: Design, workflow and effectiveness. Regen. Biomater..

[19] Rouzé l’Alzit F., Cade R., Naveau A., Babilotte J., Meglioli M., Catros S. (2022). Accuracy of commercial 3D printers for the fabrication of surgical guides in dental implantology. J. Dent..

[20] Garza-Cisneros A.N., García-Pérez M.M., Rodriguez-Guajardo W.J., Elizondo-Riojas G., Negreros-Osuna A.A. (2024). Cost-effective Solution for Maxillofacial Reconstruction Surgery with Virtual Surgical Planning and 3D Printed Cutting Guides Reduces Operative Time. Plast. Surg. (Oakv.).

[21] de Almeida E.O., Pellizzer E.P., Goiatto M.C., Margonar R., Rocha E.P., Freitas A.C., Anchieta R.B. (2010). Computer-guided surgery in implantology: Review of basic concepts. J. Craniofac. Surg..

[22] Sayed M.E., Alshehri A.H., Al-Makramani B.M.A., Al-Sanabani F., Shaabi F.I., Alsurayyie F.H., Ahmed W.M., Al-Mansour H., Jain S. (2021). Accuracy of Master Casts Generated Using Conventional and Digital Impression Modalities: Part 1—The Half-Arch Dimension. Appl. Sci..

[23] Cao R., Zhang S., Li L., Qiu P., Xu H., Cao Y. (2023). Accuracy of intraoral scanning versus conventional impressions for partial edentulous patients with maxillary defects. Sci. Rep..

[24] Rekow E.D. (2020). Digital dentistry: The new state of the art—Is it disruptive or destructive?. Dent. Mater..

[25] Mangano F., Shibli J.A., Fortin T. (2016). Digital dentistry: New materials and techniques. Int. J. Dent..

[26] de Magalhães A.A., Santos A.T. (2025). Advancements in Diagnostic Methods and Imaging Technologies in Dentistry: A Literature Review of Emerging Approaches. J. Clin. Med..

[27] Suese K. (2020). Progress in digital dentistry: The practical use of intraoral scanners. Dent. Mater. J..

[28] Revilla-Leon M., Frazier K., Da Costa J.B., Kumar P., Duong M.-L., Khajotia S., Urquhart O. (2021). Intraoral scanners: An American dental association clinical evaluators panel survey. J. Am. Dent. Assoc..

[29] Milgrom P.M., Horst J.A. (2017). The Effect of New Oral Care Technologies on the Need for Dentists in 2040. J. Dent. Educ..

[30] Ohyama H., Duong M.L., Yancoskie A.E., Smiley A.B., Syed A.Z., Reddy M.S., Bencharit S., Smiley A.Z. (2025). Challenges and Opportunities in Implementing Digital Technology in Dental Curriculum: A Review and Perspective. Cureus.

[31] van der Zande M.M., Gorter R.C., Bruers J.J.M., Aartman I.H.A., Wismeijer D. (2018). Dentists’ opinions on using digital technologies in dental practice. Community Dent. Oral Epidemiol..

[32] Sônego M.V., Neto C.L.M.M., Dos Santos D.M., Moreno A.L.D.M., Bertoz A.P.D.M., Goiato M.C. (2022). Quality of Life, Satisfaction, Occlusal Force, and Halitosis after Direct and Indirect Relining of Inferior Complete Dentures. Eur. J. Dent..

[33] Gauci M.O. (2022). Patient-Specific Guides in Orthopedic Surgery. Orthop. Traumatol. Surg. Res..

[34] Mistry R., Pisulkar S.K., Borle A.B., Godbole S., Mandhane R. (2018). Stability in complete dentures: An overview. IOSR J. Dent. Med. Sci..

[35] Jain P., Rathee M. (2020). Stability in Mandibular Denture.

[36] Bessadet M., Drancourt N., El Osta N. (2024). Time Efficiency and Cost Analysis Between Digital and Conventional Workflows for the Fabrication of Fixed Dental Prostheses: A Systematic Review. J. Prosthet. Dent..

[37] van Noort R. (2012). The future of dental devices is digital. Dent. Mater..

[38] Róth I., Hermann P., Vitai V., Rózsa N., Madléna M., Borbély J. (2023). Comparison of the Learning Curve of Intraoral Scanning with Two Different Intraoral Scanners Based on Scanning Time. BMC Oral Health.

[39] Alqahtani S.M., Chaturvedi S., Alahmari M.A., Alaleyani A.M., Alqahtani A.A., Sahal A.A., Salem M. (2024). Digital Impression (Intraoral Scanners) and Factors Affecting Its Accuracy—An Insight into Knowledge and Awareness Amongst Graduates and Clinical Practitioners. BMC Oral Health.

